# Plastic-Derived Pollutants as Emerging Modifiers of Viral Diseases

**DOI:** 10.3390/pathogens15030270

**Published:** 2026-03-03

**Authors:** Fatima Hisam, Ramina Kordbacheh, Ebenezer Senu, Spandan Mukherjee, Jon Sin, Erica L. Sanchez

**Affiliations:** Biological Sciences Department, University of Texas at Dallas, Richardson, TX 75080, USA; fatima.hisam@utdallas.edu (F.H.); ramina.kordbacheh@utdallas.edu (R.K.); ebenezer.senu@utdallas.edu (E.S.); spandan.mukherjee@utdallas.edu (S.M.); jon.sin2@utdallas.edu (J.S.)

**Keywords:** plastic pollutant, phthalates, dysregulation, viral infection, microplastics

## Abstract

Plastic pollutants, including phthalates, bisphenol A (BPA), per- and polyfluoroalkyl substances (PFAS), and microplastics (MPs), are increasingly recognized as emerging environmental cofactors that intersect with infectious disease dynamics. These compounds, once considered inert, can alter immune function, reshape host–pathogen interactions, and directly influence viral survival and transmission. In this review, we compile current evidence on the chemistry, environmental occurrence, and biological activity of major plastic-associated pollutants with emphasis on their role in viral infections. Phthalates such as di(2-ethylhexyl) phthalate (DEHP) and its metabolite MEHP modulate innate immune signaling and have been shown to exacerbate infections, including Dengue and Coxsackievirus B3. Other DEHP-like phthalates, such as dibutyl phthalate (DBP), exhibit consistent infection-enhancing effects, while high molecular weight or cyclical phthalates such as polyvinyl acetate phthalate (PVAP) display conflicting results in their modulation of viral infections. BPA, widely detected in human tissues, acts through endocrine and immune disruption, worsening viral myocarditis, and altering influenza outcomes. PFAS, persistent “forever chemicals,” reshape adaptive immune responses and are associated with increased susceptibility, viral persistence, or severity of infection of herpesvirus (HCMV, EBV, HSV-1), hepatitis virus, and influenza infection. Microplastics represent a distinct risk by acting as physical carriers for viruses and bacteria, stabilizing viral RNA, enhancing host cell uptake, and skewing immune responses. Together, these pollutants extend beyond toxicology into virology, providing novel insights into how environmental exposures converge with viral pathogenesis. We highlight mechanistic advances and critical knowledge gaps and propose future directions for integrating environmental health and infectious disease research.

## 1. Introduction

Plastics have become ubiquitous in modern life, with global production surpassing 460 million tons annually and projected to triple by 2060 [1]. Their widespread use, environmental persistence, and breakdown into diverse chemical and particulate forms mean that humans are exposed to plastic byproducts through air, water, food, and direct contact across the lifespan. Historically, the health impacts of plastic pollutants were framed around endocrine disruption and carcinogenesis, as well as associations with chronic diseases including asthma, allergy, metabolic syndrome, and cardiovascular dysfunction. Yet, a growing body of research reveals a less explored but highly consequential dimension of their ability to modulate infectious diseases, particularly viral infections.

Among the studied plastic-associated chemicals are phthalates, bisphenols, and per- and polyfluoroalkyl substances (PFAS), along with particulate microplastics (MPs). Plastic-associated pollutants exist across multiple classes, each with distinct chemistries and biological activities. Phthalates are widely used as plasticizers to impart flexibility and durability to polymers such as polyvinyl chloride (PVC), with di(2-ethylhexyl) phthalate (DEHP) being among the most extensively produced and studied members [2]. Because phthalates are not covalently bound to plastics, DEHP and its metabolites readily leach, leading to human exposure via ingestion, inhalation, and dermal contact [3]. Beyond well-established reproductive toxicity, epidemiological and experimental data link phthalate exposure to asthma, metabolic disorders, and immune dysregulation [4]. Phthalate exposure, implicated in metabolic disturbances such as insulin resistance, obesity, and dyslipidemia, may suggest interference with lipid and glucose homeostasis. At the immunological level, phthalates may modify cytokine production and alter the behavior of immune cells, including macrophage and lymphocyte activity, collectively contributing to immune imbalance and inflammation. Similarly, phthalates increased susceptibility to viral infections [5]. While best known for reproductive toxicity, emerging evidence shows that phthalates also impair antiviral immunity.

Moreover, bisphenol A (BPA), a common monomer in polycarbonate plastics and epoxy resins, disrupts endocrine and immune signaling. BPA is present in bottles, tableware, cookware, medical devices, printing inks, and flame retardants [6]. Mechanistically, BPA activates estrogen receptor α and β, promoting carcinogenesis [7], and also binds to androgen and thyroid hormone receptors, perturbing downstream immune and metabolic pathways [8,9].

In addition, per- and polyfluoroalkyl substances (PFAS), persistent “forever chemicals” found in nonstick and water-repellent products, accumulate in human serum and tissues [10]. PFAS consist of highly fluorinated carbon chains, either linear or branched, and are used widely in cookware, detergents, pesticides, and floor waxes [11]. The most well-known members are perfluorooctanesulfonate (PFOS), perfluorooctanoic acid (PFOA), perfluorohexanesulfonate (PFHxS), and perfluorononanoic Acid (PFNA), which are exposed to humans via contaminated water, food intake, and house dust [12]. Emerging epidemiological and experimental evidence links PFAS exposure to suppressed T- and B-cell responses, reduced vaccine efficacy, and increased intensity of viral infections [13,14]. Reduced vaccine efficacy in this context refers to diminished antigen-specific immune responses following immunization, most reflected by lower circulating IgG antibody titers and reduced seroconversion rates. Thus, this study among participants aged 12–90 years who received the mRNA COVID-19 vaccine demonstrated that elevated serum PFAS concentrations are associated with weaker antibody responses to the vaccines [14]. Mechanistically, PFAS exposure impaired B-cell differentiation, altered germinal center formation, and reduced plasma cell survival, all of which are essential for generating high-affinity, long-lived antibody responses. In parallel, PFAS may disrupt T follicular helper (Tfh) cell function and cytokine signaling required for effective B-cell activation and class switching. Reduced vaccine efficacy implies not only lower immediate antibody titers but also potential impairment of immune memory formation, raising concerns about sustained protection against both vaccine-preventable and naturally acquired viral infections. Given the long biological half-lives of several PFAS compounds, chronic exposure may result in prolonged immune modulation, with implications for population-level immunity and outbreak vulnerability. PFAS bioaccumulate with half-lives measured in years, and are further implicated in cardiovascular and metabolic dysfunction [15], liver and immune dysfunction [16,17], and even cancer [18].

A particularly concerning development is the rise of microplastics (MPs), synthetic polymer fragments < 5 mm in size, originating from industrial production and environmental degradation of larger plastics [19]. MPs are now detected in air, food, water, and human tissues, including the lung and placenta [20]. Beyond their toxicological effects, MPs have been reported to interact directly with microbes and viruses. Emerging laboratory and computational studies show that MPs can adsorb virions, stabilize viral RNA, and promote viral uptake into host cells [21,22].

Despite accumulating evidence across these pollutant classes, current knowledge remains scattered and methodologically heterogeneous. Studies vary widely in exposure assessment, biological models, and outcome measures, limiting cross-comparison and mechanistic synthesis. Moreover, most investigations focus on single chemicals in isolation, whereas real-world exposure occurs as complex mixtures over prolonged periods. A comprehensive, categorized integration of existing evidence is therefore needed to clarify how plastic-associated pollutants intersect with viral pathogenesis. In this review, we synthesize chemical, immunological, mechanistic, and epidemiological data across the major pollutant categories, phthalates, bisphenols, PFAS, and microplastics, to evaluate their potential roles as environmental cofactors of viral infection outcomes. By bridging environmental toxicology and virology, this review highlighted mechanistic pathways, identified critical gaps, and provided a framework for investigating how global plastic pollution may influence infectious disease dynamics in the context of future viral pandemics and public health.

## 2. Literature Search Strategy

The MEDLINE (PubMed) and the Cochrane Library were used as the primary databases searched for articles published in English up to date. PubMed was further checked for relevant MeSH terms. Additional broader databases such as Google Scholar, Web of Science, and Scopus were also searched for any additional articles. The search strategy developed include “Phthalate” OR “Phthalates” OR “Diethylhexyl phthalate” OR “Di-n-butyl phthalate” OR “Diisononyl phthalate” OR “Diisodecyl phthalate” OR “Benzyl butyl phthalate” OR “DEHP” OR “DBP” OR “DINP” OR “DIDP” OR “BBP” OR “Bisphenol A” OR “BPA” OR “Microplastics” OR “Plastic pollutants” [All Fields]; “Infectious disease” OR “Infectious diseases” OR “Virus” [All Fields]; “UpToDate (30 December 2025)” [Publication Date]; #1 AND #2 AND #3 AND #4. The search strategy used was modified according to the respective databases. Studies referenced in all included studies were also retrieved to obtain further related studies. The analyses of search results followed the Cochrane guidelines for reporting the review.

## 3. Inclusion and Exclusion Criteria

The review considered studies reporting on the major pollutant categories: phthalates, bisphenols, PFAS, and microplastics to evaluate their potential roles as environmental co-factors or modifiers of viral infection outcomes. Studies conducted to assess these impacts using models including humans, mice, cell lines or from environmental-related factors were included. Articles on epidemiological, clinical, human, and in-vitro related models were included. However, articles mainly centered on animal infections and/or case reports, meeting reports, and conference papers were excluded from the main synthesis of the results.

## 4. Phthalates

**Figure 1:**
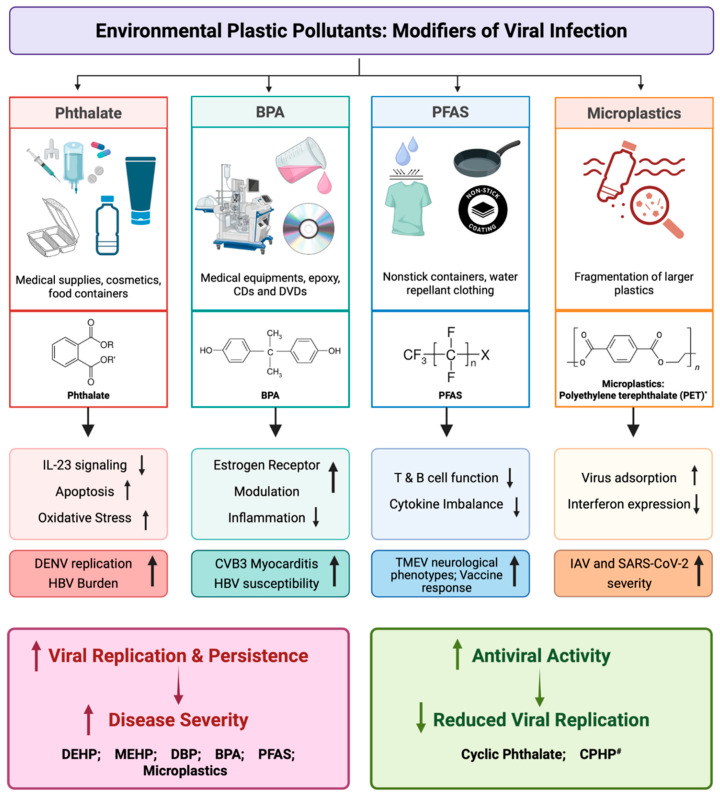
Schematic representation of plastic pollutants and their links to virus-mediated human diseases. Industrial phthalates (DEHP, MEHP, DBP) impair innate and adaptive antiviral responses, increasing viral replication and disease severity during infections such as DENV, HBV, and CVB3. BPA alters estrogen receptor signaling and inflammation, exacerbating CVB3 myocarditis, and increasing HBV susceptibility. PFAS disrupt T- and B-cell function and cytokine balance, contributing to impaired vaccine responses and worsened outcomes during viral infection. Microplastics adsorb and stabilize viruses, suppress interferon signaling, and skew host immune responses, enhancing disease severity in IAV and SARS-CoV-2 models. In contrast, cyclic phthalates, natural phthalate (CPHP), and high molecular weight phthalate derivatives (CAP, HPMCP, and PVAP) exhibit antiviral activity by interfering with host lipid metabolism and viral replication. Arrows indicate directionality of effect. * Polyethylene terephthalate (PET) is shown as an example of a microplastic. ^#^ CPHP denotes a natural phthalate.

Phthalates represent a diverse family of chemicals used across countless industrial products, making human exposure unavoidable [3]. It has become clear that these compounds do far more than simply soften plastics, and they can interact with immune pathways, metabolic systems, and even viral infection dynamics [23]. We have described different groups of phthalates and their link to virus infection pathogenesis in the following paragraphs (Table 1, Figure 1).

Di(2-ethylhexyl) phthalate (DEHP) is a common industrial plasticizer that, upon entering the body, is rapidly metabolized to mono(2-ethylhexyl) phthalate (MEHP) [2,23]. Because both are lipophilic, they can leach easily from plastics, leading to widespread human exposure through food contact materials, indoor air, and certain medical devices [3]. Over the past decade, studies have shown that DEHP and MEHP can alter immune function, potentially altering how the body responds to viral infections. In Dengue virus (DENV) infection, exposure to MEHP was shown to enhance viral replication by impairing macrophage antiviral function. Post-cytotoxicity screening from 1 nM up to 1 mM MEHP, the key treatment doses used in this study were 50 nM, 100 nM, and 200 nM MEHP, which were below cytotoxic thresholds. The investigators showed that MEHP suppressed IL-23 production and IL-23-dependent signaling pathways in macrophages, resulting in reduced induction of antiviral effector responses that normally limit early DENV replication. This effect was consistently observed in macrophages derived from patient samples as well as in vitro infection models [5]. By attenuating macrophage-mediated innate immune signaling, MEHP creates a cellular environment that favors viral replication during the early stages of infection. Another study using human cell lines suggests the impact may not be limited to DENV but that DEHP exposure also worsens outcomes in Coxsackievirus B3 (CVB) infection as well [24]. Based on this study, CVB infection increased after 40 µg/mL DEHP exposure across different cell types (HeLa, iBECs, Caco-2, HL-1), leading to increased viral particle release and alterations in the CVB-induced interferon signaling pathway by suppressing antiviral immunity via inhibiting interferon regulatory factor 7 and enhancing interferon-induced transmembrane proteins. Mechanistic work suggests several possible routes for these effects. MEHP can suppress IL-23 production in macrophages [5]; DEHP and MEHP can signal through the PPARγ pathway, altering dendritic cell development and modifying immune function [35]; and broader disruptions to immune cell populations, potentially linked to microbiome changes, may leave the host more vulnerable to infection [36]. The scope of these effects appears to depend on the dose, timing, and specific metabolites involved [37]. Although current evidence is compelling, human studies directly linking measured DEHP/MEHP levels to worse outcomes for specific viral infections remain limited [5,24].

Moreover, DEHP and MEHP can induce oxidative stress, activating or repressing key pathways such as NF-κB and MAPK, which, in turn, regulate inflammatory gene expression [38]. Another research study investigated postnatal exposure to DEHP in newborns. Significantly higher urinary phthalate levels were observed in newborns receiving intravenous infusions because of respiratory distress. Additionally, it was shown that humoral immunity can also be weakened by reduced IgM production after vaccination in infants with early-life DEHP exposure, potentially due to B-cell activation failure or reduced T-cell recruitment [39].

Together, these studies demonstrate that phthalates exert diverse and context-dependent effects on viral infection outcomes by modulating host immune and metabolic pathways rather than acting uniformly as proviral or antiviral agents. DEHP and its metabolite MEHP function primarily as immune disruptors, impairing innate antiviral signaling, altering interferon pathways, and weakening vaccine-induced or early antiviral responses, thereby increasing susceptibility to infection or disease severity. These observations identify DEHP and MEHP as environmental immune disruptors capable of directly impairing antiviral defense mechanisms, emphasizing the importance of studying their contribution to viral disease susceptibility in humans.

## 5. Other Phthalates

Aside from DEHP, other phthalate-containing compounds like dibutyl phthalate (DBP), cyclic phthalates, and 2″-(methoxycarbonyl) 5″-methylpentyl 2′-methylhexyl phthalate (CPHP) have shown conflicting effects on viral infection (Table 1, Figure 1). DBP is a plasticizer used in many applications similar to DEHP, such as plastic packaging, and can accumulate in indoor environments with consistent exposure [40]. In a human study, DBP was found to accumulate in the liver tissues of patients with Hepatitis B virus (HBV) infection and to impair hepatic T cells by inducing apoptosis and ferroptosis. In the process, 100 mg/kg/day DBP exposure caused an increase in hepatic viral load. Long-term data between 2003–2018 also revealed a correlation between urinary phthalate metabolites such as MnBP (DBP) and MEHP (DEHP), and the presence of higher serological concentrations of HBV surface antigens, HBsAg (by MnBP and MEHP) and HbcAb (by MnBP) [25]. Conversely, cyclic phthalates are cyclical structures containing multiple copies of the same phthalate compound and were identified as Liver X receptor (LXR) antagonists from a chemical library. Cyclic phthalates, compound 1 (ester dimer) and compound 2 (ester trimer), acted as antagonists of LXR activity in Huh-7 cells (human liver carcinoma cell line) and subsequently lowered Hepatitis C viral (HCV) production. However, compound 1 at 40 μM reduced the viral RNA of SARS-CoV-2 while significantly reducing lipid droplet formation. Alternatively, compound 2 showed no significant anti-SARS-CoV-2 activity at ≤20 µM but was toxic at 40 µM in the test cells (VeroE6/TMPRSS2) [26]. The structure of cyclic phthalates may cause differential activity from linear phthalates because DINP (diisononyl phthalate) did not significantly inhibit HCV replication up to 30 µM concentrations [26]. Similarly, 2″-(methoxycarbonyl) 5″-methylpentyl 2′-methylhexyl phthalate (CPHP) is a natural phthalic acid ester bearing structural resemblance to DEHP. CPHP was found to reduce viral titers in Vero and LLC-MK2 cells infected with DENV2 and Human parainfluenza virus type 3 (hPiV3) strains. CPHP was also found to be less cytotoxic and more effective, with an EC_50_ of 29.4 µM, at lowering post-infectious viral titers of hPiV3 than the established antiviral control BCX 2798. Against Chikungunya virus (CHIKV), CPHP displayed antiviral activity similar to Ribavirin [27]. This study was the first to show the antiviral activity of phthalates against (+) and (−) strand RNA viruses. Collectively, these observations on the differential effects of phthalate plasticizers on various viral infections further highlight the importance of understanding the diversity of cellular mechanisms that phthalates alter, which ultimately influence viral infection.

Much of the current viral research on phthalates focuses on the pollutant activity of phthalates used in industrially manufactured plastics as plasticizers. However, high molecular weight phthalate derivatives of cellulose, like cellulose acetate phthalate (CAP, ~0.1–1 mg/mL) and hydroxypropyl methylcellulose phthalate (HPMCP, ~0.05–0.5 mg/mL), have historically been explored for their antiviral activity against HIV along with herpesviruses and, when formulated into micron-sized particles (micronized), inactivate HIV-1, HSV-1, HSV-2, and Human Cytomegalovirus (HCMV) [41]. The attenuation of HIV-1 infection was traced to CAP, applied at 0.1–1.0 mg/mL levels, directly binding to HIV-1 virions, with the envelope glycoprotein gp120 reducing the activity of coreceptors CXCR4 and CCR5 [42]. Viral attenuation of HSV-2 by 2% (*w*/*w*) CAP vaginal gel was also confirmed in vivo with a mouse model [43]. Further, electrospun CAP fibers loaded with tenofovir (TNF), when used at ~70% CAP, ~30% TFV with 10–20 mg/mL extract effective were found to neutralize HIV-1 virions after 1 h of incubation and investigated as an intra-vaginal method of anti-viral drug delivery [44]. Various versions of HPMCP and Polyvinyl acetate phthalate (PVAP) were shown to have antiviral activity against HSV-1 and HSV-2. Exposure to PVAP reduced viral entry of HSV-1 in a dose-dependent manner, but not HSV-2, into uninfected HCE and HeLa cells. However, pretreatment of uninfected cells with HPMCP-55S reduced viral entry, and exposure to 100–200 µg/mL HPMCP-55S reduced the number of infectious HSV2 virions in whole cell lysate from infected HCE and HeLa cells. In their nanoparticular forms (NP), both PVAP and HPMCP-55S showed a greater efficacy of their respective antiviral activity. Interestingly, PVAP NPs displayed synergistic activity with other known anti-herpetics like AVC, Tenofovir, and Tenofovir Disoproxil Fumarate (TDF), making previously ineffective concentrations of all three drugs display significant antiviral activity [45]. The antiviral promise of phthalate-based compounds is still not completely understood, and the creation of new antiviral drugs will need to be based on a better understanding of the phthalate–virus interaction.

In conclusion, these structurally distinct phthalates exhibit direct antiviral activity against multiple RNA viruses by interfering with host lipid metabolism or viral entry. Additionally, high-molecular-weight phthalate derivatives developed for pharmaceutical use demonstrate potent virucidal and entry-inhibitory properties, underscoring the importance of chemical structure in determining biological outcomes. Together, these findings highlight that phthalates represent a chemically heterogeneous class whose effects on viral infection range from immunotoxicity to antiviral activity, emphasizing the need to evaluate individual compounds, exposure context, and mechanisms when assessing their impact on viral pathogenesis and therapeutic potential.

## 6. Bisphenol A (BPA)

Bisphenol A (BPA) is a chemical monomer primarily found in the production of polycarbonate plastics, like reusable plastic bottles, feeding bottles, and storage containers, as well as in the production of epoxy resins for coating food cans and water pipes [6]. It is also widely used in everyday products, such as inks, textiles, toys, CDs, medical devices, or dental sealants [46,47]. Major sources of BPA exposure to humans are through diet, air, water, and soil, posing toxic, mutagenic, and carcinogenic effects in humans and other species [48,49]. Exposure to BPA during viral infections can dysregulate the immune system and other host cell responses [50] (Table 1).

A study done on Coxsackievirus B3 (CVB3) showed that human-relevant doses of BPA (5 µg BPA/kg body weight (BW)) exposure in BALB/c mice significantly increased viral myocarditis when compared to mice not exposed to BPA [30]. Interestingly, BPA exposure did not alter viral genome levels significantly during CVB3 infection. However, 5 µg/kg BW BPA exposure had a significant prevalence of pericarditis, inflammation of the pericardium, and increased cardiac CD4+ T cells, cardiac mast cell numbers, and pericardial mast cell degranulation. Interestingly, during viral myocarditis in mice exposed to BPA, ER alpha is decreased, and ER beta is increased, suggesting that BPA interferes with cardiac inflammation by dysregulation of ERs. Furthermore, inflammatory mediators like IFN gamma, IL-17A, TLR-4, caspase-1, and IL-1 beta are also significantly increased in BPA-exposed mice suffering from myocarditis. These findings suggest that there is increased viral myocarditis and immune dysregulation after BPA exposure. Interestingly, an epidemiologic study was conducted to link environmental BPA exposure to immune response following HBV vaccination in a large, nationally representative U.S. sample [28,51]. An increase in the odds for susceptibility to HBV infection was observed with an increase in urinary BPA concentration. While the suggested result does not prove attenuation of adaptive immune responses associated with BPA exposure, this study asks a novel question of the effects of chemical exposure on vaccine-specific immunity. Additionally, research conducted on pregnant mice (dams) found that exposure to BPA did not change the body weight of the dams or their offspring, with no noted differences in neonatal survival, litter size, or sex of the offspring [29]. Furthermore, lung inflammation in adult mice that were exposed to BPA developmentally had significantly less severity by 7 days post infection with Influenza A virus (IAV). Interestingly, inflammatory molecules like IP-10 (CXCL10) and inducible nitric oxide (iNOS) had significantly reduced gene expression at 7 days post-IAV infection, while expression levels of chemokine, RANTES (CCL5), and iNOS were significantly decreased at 10 days post-IAV infection. It is interesting to note that while toll-like receptor (TLR)-3, interferon (IFN)-*β*, and interleukin (IL)-6 were altered by IAV infection, they were not modulated due to developmental BPA exposure in mice. Furthermore, neither adaptive immune response nor memory immune response was influenced by 7 to 9 days post-IAV infection. These results collectively suggest that anti-viral immune responses are not altered during IAV infection due to developmental exposure to BPA.

In conclusion, exposure to BPA can affect antiviral immune responses; however, the impact varies depending on the virus and exposure conditions (Figure 1). BPA is linked to elevated HBV susceptibility and exacerbates CVB3-induced myocarditis, indicating immunological dysregulation. These results demonstrate that the effect of BPA on viral immunity varies depending on the type and timing of exposure and is virus-specific.

## 7. Perfluoroalkyl Substances (PFAS)

Per- and polyfluoroalkyl substances (PFAS) are highly fluorinated carbon chains that do not break down easily and are commonly found in nonstick and water-repellent products like cookware, detergents, pesticides, and many others [51]. PFAS exposure in humans occurs via water, food, and air, with significant serum levels reported globally leading to bioaccumulation correlated with level of exposure, such as higher serum concentrations in construction workers who are routinely exposed to PFAS in building materials [52]. PFAS exposure also leads to toxic and carcinogenic symptoms in humans and other animals [53]. PFAS exposure during viral infection may lead to impaired immune response, reducing vaccine effectiveness as demonstrated by prenatal PFAS exposure correlating with reduced rubella, diphtheria, and tetanus antibodies in children and even triggering chronic inflammation, such as PFAS exposure correlating to increased biomarker levels for rheumatoid arthritis [54,55] (Table 1, Figure 1).

A recent serological study done on the data collected from the National Health and Nutrition Examination Survey (NHANES) observed the effects of environmental PFAS exposure during persistent infections in humans [31]. The serum data included individuals of 12–49 years old with the exact PFAS measurements in the presence of infection of one of the persistent pathogens including HCMV, Epstein Barr virus (EBV), hepatitis virus types C and E (HCV, HEV), human immunodeficiency virus (HIV), herpes simplex virus types 1 and 2 (HSV-1, HSV-2) and two bacterial species. The four highly detectable PFAS molecules were perfluorooctanesulfonate (PFOS), perfluorooctanoic acid (PFOA), perfluorohexanesulfonate (PFHxS), and perfluorononanoic acid (PFNA). Among adolescents, who exhibit higher EBV prevalence, HSV-1 infection was associated with elevated PFOS levels. In contrast, adult serum-associated HSV-1-infected individuals had higher PFAS concentrations, with increased PFOS and PFOA associated with higher pathogen burden. Interestingly, the serum concentrations of PFOS, PFOA, and PFHxS are found to be reduced over the year, which could be associated with the replacement of PFAS chemicals with newer chemical structures like GenX.

Another study was performed on exposure to a mixture of PFAS chemicals in mice during influenza A virus (IAV) infection [32]. Either a binary mixture (BM) of PFOS and PFOA or a quaternary mixture (QM) of PFOS, PFOA, PFHxS, and PFNA was used to study the immunotoxicity of PFAS mixtures in the C57Bl/6 mouse model with IAV infection. Mice treated with BM post-infection showed a significant decrease in body weight, while control mice treated with BM had no body weight changes. Notably, BM significantly reduced CD8+ T cells, cytotoxic T lymphocytes, CD4+ T-cells, and activated (CD44hi) CD4+ T-cells. However, QM treatment did not show significant differences in T cell responses to IAV. While QM significantly reduced B-cells (CD19^+^, CD3^−^) in mediastinal lymph nodes, neither of the PFAS mixtures affected the germinal center B cells and the number of plasma cells during IAV infection. Interestingly, the BM and QM were shown to differentially affect the adaptive immune response to IAV infection by a significant reduction in relative IAV-specific IgM by BM and no significant changes when exposed to either mixture. This study compared the effects of two different PFAS mixtures on the adaptive immune response to infection with a common respiratory pathogen. The analysis revealed that BM affected the T-cell response and production of IAV-specific IgM, while the QM affected aspects of the B-cell response.

Another group studied the effects of PFOA exposure during Theiler’s murine encephalomyelitis virus (TMEV) infection in C57BL/6J (B6) mice for the first time, and demonstrated that IL-4 and IL-13 were suppressed during PFOA, hence creating an imbalance of cytokines [33]. TMEV, a neurotropic RNA virus, is used as a model to depict human neurological symptoms linked with viral infections [56]. Additionally, neurological phenotypes were observed in otherwise TMEV infection-resistant C57BL/6J (B6) mice, where piloerection was significantly higher in the PFOA+TMEV infection group when compared to non-infected PFOA-exposed mice. Other disturbed neurological phenotypes, including limb weakness, limb paralysis, retropulsion, and delayed righting reflex, were observed in TMEV-infected mice regardless of PFOA exposure, while the frequency of these phenotypes was greatly increased during infection and exposure to 70 ppt PFOA. Seizure score 3 on a scale of Racien seizure scale was more significantly observed in TMEV-infected mice exposed to 70 ppt PFOA. TMEV RNA expression levels in the hippocampus and thoracic spinal cord had no significant differences in TMEV-infected mice regardless of 70 ppt PFOA exposure, which suggested no direct interaction with viral mechanisms. PFOA exposure to mice altered Th1 cytokines and suppressed Th2 cytokines when compared to control groups, and PFOA-induced cytokine alterations influenced TMEV RNA levels and seizure frequency. These results demonstrate the complex roles of immune responses due to dual exposures to neurological viruses and immunotoxic compounds, PFOA. These results also suggest PFOA induced altered cytokine and chemokine production; however, differential doses and mixtures of PFAS compounds are required.

A recent cross-sectional study conducted across three U.S. communities, including 330 participants showing no history of COVID-19 diagnosis, with elevated PFAS exposure, investigated five serum PFAS concentrations in relation to anti-spike SARS-CoV-2 IgG levels following COVID-19 vaccination [34]. Despite well-documented associations between PFAS and immune dysregulation, particularly in pediatric populations, the study found no consistent evidence that higher PFAS burdens were associated with diminished vaccine-induced antibody responses in adults. Isolated associations between individual PFAS compounds and antibody levels were observed within specific communities. Only in the community from the Lower Cape Fear River region had a statistically significant increase of 0.31% anti-S IgG per 1% increase in perfluoroheptane sulfonic acid (PFHpS). However, these effects lacked consistency and did not support a generalized immunosuppressive pattern. These findings underscore the complexity of PFAS-immune interactions and highlight potential age, exposure, or context-dependent differences in vaccine responsiveness.

Current research demonstrates that PFAS exposure can disrupt antiviral immunity during viral infection. Human serological data show higher PFAS levels with increased pathogen burden during persistent viral infections, while animal studies demonstrate that specific PFAS mixtures can impair T-cell or B-cell responses during IAV infection. Additionally, PFOA exposure worsens neurological symptoms during TMEV infection by altering cytokine balance without affecting viral load. Importantly, recent vaccine studies in adults with elevated PFAS exposure show no robust attenuation of SARS-CoV-2 vaccine-induced antibodies, highlighting potential age and exposure-specific differences in susceptibility. Together, these findings underscore the need to move beyond single-compound paradigms toward mixture-based, longitudinal, and mechanistic approaches to fully define how PFAS exposures intersect with viral pathogenesis and vaccine-mediated immunity.

## 8. Microplastics

Microplastics (MPs) continue to be a major public health concern due to their increasing presence in most ecosystems. The term “microplastics” was first used over 20 years ago during investigations of maritime plastic contamination in the United Kingdom [57]. They are synthetic polymer particles to be <5 mm generated intentionally, such as primary MPs from cosmetic microbeads, industrial pellets, or by fragmentation/abrasion of larger plastics, such as secondary MPs including fibers from textiles, tire wear. The amount of plastic produced globally has increased significantly over the last 70 years [58]. An estimated 460 million tons of plastic products were produced worldwide in 2019, with just 9% of these being recycled, and it is projected that plastic production will rise to 1.2 billion tons by 2060 [1]. The most common polymer chemistries detected in air, water, food, and biota include polyethylene (PE; (C2H4)n), polypropylene (PP; (C3H6)n), polystyrene (PS; (C8H8)n), polyvinyl chloride (PVC; (C2H3Cl)n), polyethylene terephthalate (PET; (C10H8O4)n), and poly(methyl methacrylate) (PMMA; (C5H8O2)n) [59,60]. Humans are exposed to microplastics via ingestion and inhalation in ambient air, water, and food. These particles can be detected in multiple human tissues and organs, including the lung, placenta, and blood, highlighting widespread exposure and raising concerns regarding potential health effects [1]. Converging data suggest that MPs can dysregulate immunity and inflammation, with epidemiologic and toxicologic work linking respiratory, gastrointestinal, and reproductive outcomes. In infectious disease models, MPs can modulate diverse host responses to facilitate viral infections via some of the following mechanisms (Table 1, Figure 1).

Microplastics act as physical carriers of viruses and bacteria: One study, using T4 bacteriophage and polystyrene microplastics, showed in vitro that up to 98.6 ± 0.2% of dosed viruses adsorbed to MPs and adsorption depended on size and surface functionalization [61]. In this study, both fluorescence-labeled confocal microscopy and Fourier-transform infrared spectroscopy confirmed that the virus may effectively adsorb onto microplastics. Zeta potential characterization indicates that electrostatic interaction is the main adsorption mechanism linked to virus adsorption. Both pristine and UV-aged microplastics prolonged viral infectivity even at elevated temperatures, demonstrating carrier-protection effects. Previous reviews have also raised concerns about microplastics being able to rapidly develop biofilms harboring bacteria, fungi, algae, and viruses, creating mobile microbial consortia that differ from the surrounding water or air and can include pathogens and antibiotic-resistance genes [62,63]. Thus, MPs can shuttle infectious particles through air and water, concentrate them at air–liquid interfaces and surfaces, and potentially extend environmental persistence windows relevant to transmission.

Moreover, MPs promote viral attachment, cellular uptake, and viral persistence on host cells. In A549 cells infected with H1N1 influenza A virus (IAV), polystyrene MPs (PS-MPs) significantly increased viral infection [21]. This study observed virus enrichment on PS surfaces and enhanced viral entry via endocytosis of MP–virus complexes. PS-MPs decreased antiviral defenses, including the interferon-inducible transmembrane protein IFITM3 and upstream RIG-I/IRF3/TBK1 signaling, thereby reducing IFN-β expression and impairing innate responses [26]. Another study also found airborne MPs could bind viruses such as SARS-CoV-2 aerosols, prolong viral persistence, and enhance the likelihood of respiratory entry, thereby linking microplastic pollution directly with viral exposure risks in urban environments [22].

Furthermore, in a mouse Omicron BA.5 SARS-CoV-2 infection model, lung-deposited MPs suppressed early innate immune pathways 2 days post-infection (dpi) and amplified pro-inflammatory signatures by 6 dpi, despite no significant change in lung viral titers, indicating host-response skewing that could worsen disease phenotypes or recovery [64]. In addition, MPs interact with viral RNA/proteins to enhance exposure and infectivity: Molecular-dynamics simulations show that multiple MP chemistries (PE, PP, PS, PVC, PET) exhibit favorable binding to a SARS-CoV-2 RNA fragment through a combination of hydrophobic and electrostatic interactions; the interaction affinity for SARS-CoV-2 RNA exceeded that for SARS-CoV-1 and HBV fragments, across temperatures and in water/vacuum [65]. Such interactions could stabilize RNA and alter transport. Thus, at the nanoscale, aged, roughened, or biofouled MPs present heterogeneous charge patches and functional groups that can nucleate RNA/protein adsorption or corona formation, with plausible consequences for particle-mediated co-transport, shielding from degradation, and receptor-bypassing uptake pathways.

Accumulating evidence indicates that MPs can exacerbate viral exposure and disease risk by acting as both physical carriers of pathogens and biological modifiers of host immune responses. MPs increase opportunities for host contact and transmission by adsorbing and stabilizing viruses, while at the cellular level, they promote viral attachment and uptake while suppressing innate antiviral defenses, skewing host responses toward enhanced infection or inflammation without necessarily altering viral load. In vivo studies further demonstrate that MP exposure can dysregulate early immune responses and amplify inflammatory pathology during viral infection. Collectively, these findings suggest that widespread MP pollution may represent an underappreciated environmental cofactor that modulates viral infectivity, host immunity, and disease severity.

## 9. Microplastic-Virus Interface Chemistry

In biological fluids, proteins, surfactants, lipids, and microbial products adsorb rapidly onto MPs, changing particle size/charge (“corona”) and modulating cell interactions, including enhanced phagocytic uptake (Figure 1). Depending on the MP’s size/surface area, chemical makeup, and surface charge, its surfaces can serve as solid scaffolds for bacteria, viruses, and different biomolecules (including LPS, allergens, and antibiotics) [66]. The immune system can effectively recognize and eliminate pathogens, foreign substances, and abnormal molecules through phagocytosis initiated by pattern recognition receptors [66]. However, associating with MPs can alter the physical, structural, and functional properties of microorganisms and biomolecules, altering how they interact with the host immune system (specifically, innate immune cells) and, most likely, the characteristics of the innate/inflammatory response that follows. For instance, in the lung, MPs can interact with pulmonary surfactants and generate reactive oxygen species (ROS), with implications for barrier integrity during viral infection [67]. Therefore, the microplastic association fundamentally redefines the immunological presentation of pathogens and biomolecules via the formation of a protein corona, critically mediating innate immune recognition and the ensuing inflammatory response.

## 10. Links to Human Disease Pathogenesis

Accumulating evidence demonstrates that plastic-associated pollutants do not act in isolation but intersect directly with human disease pathogenesis. The strongest links emerge in cardiovascular, respiratory, hepatic, and immune-related disorders, where viral infections and pollutant exposures converge. Phthalates, particularly DEHP and MEHP, amplify infection severity by disrupting interferon signaling and macrophage-derived cytokine responses [5,24]. CVB3, a major etiological agent of myocarditis, exploits these disrupted pathways to enhance viral replication and viral persistence, highlighting how environmental exposures can compound viral cardiac pathogenesis (Figure 1).

Moreover, BPA exposure has been associated with dysregulated pulmonary immune responses, exacerbating influenza A outcomes [29,68]. Similarly, microplastics (MPs) directly modulate lung immunity: in murine models of SARS-CoV-2 infection, MPs suppressed innate antiviral responses while driving inflammatory signatures resembling cytokine storm [64]. Airborne MPs have further been shown to bind SARS-CoV-2 RNA, linking environmental pollution to viral persistence in aerosols and heightened respiratory exposure risks [22]. These findings point to a novel intersection between pollution exposure and viral pneumonia pathogenesis.

In addition, dibutyl phthalate (DBP) impairs hepatic T-cell function and accelerates hepatitis B virus (HBV) progression, driving immune exhaustion and collapse of the hepatic microenvironment [25]. PFAS compounds, widely detected in serum, have been linked to disrupted lipid metabolism and immunosuppression, which may worsen viral hepatitis and interfere with vaccine-induced immunity [69].

Across pollutants, a recurring theme is immune suppression and skewing. PFAS exposure has been associated with diminished T- and B-cell responses, lower antibody titers following vaccination, and higher burdens of chronic viral infections [13]. Microplastics prolong viral persistence by stabilizing virions and disrupting autophagic flux, pathways increasingly recognized as critical for antiviral defense [21,61]. These mechanisms suggest that chronic exposure to pollutants may weaken antiviral immunity and predispose individuals to severe or prolonged infections. Taken together, the convergence of viral pathogens with phthalates, BPA, PFAS, and MPs provides a mechanistic basis for their contribution to infectious disease pathogenesis (Figure 1), with clinical implications ranging from worsened myocarditis to impaired vaccine responses.

## 11. Discussion

The evidence synthesized in this review challenges traditional views of plastics as passive contaminants, reframing them as active participants in infectious disease ecology. Across chemical classes, plastic-associated pollutants converge on a limited set of mechanisms such as immune dysregulation, lipid and metabolic reprogramming, and direct interactions with viral particles that facilitate viral infection, persistence, and pathogenicity. Despite distinct chemistries, phthalates, BPA, PFAS, and MPs share common outcomes. Phthalates and BPA disrupt cytokine and interferon signaling, undermining innate antiviral defenses [5,29]. PFAS suppresses adaptive immunity, lowering vaccine efficacy and antiviral responses [14]. MPs act both physically and biologically: binding viral particles, stabilizing RNA, and skewing host immune pathways [21,64]. These overlapping mechanisms suggest that pollutant exposures may synergize, producing cumulative impacts on viral pathogenesis.

Moreover, the ubiquity of plastics in air, water, and food creates continuous, low-level human exposure across populations with environmental and public health implications. The finding that airborne MPs can carry SARS-CoV-2 RNA [22] raises urgent questions about their role in viral transmission dynamics, particularly in densely populated urban centers. Furthermore, pollutants like PFAS accumulate in serum with half-lives measured in years, representing long-term modulators of host immunity. These exposures could partly explain the variability in viral disease outcomes, particularly for pathogens whose severity and clearance are highly influenced by host immune responses, such as influenza, HBV, and SARS-CoV-2.

Inhalation of airborne particles occurs across a range of environmental exposures beyond microplastics, including endogenous detritus such as human skin flakes and biological allergens like house dust mite fecal matter. Microplastics are persistent synthetic polymer particles that can deposit in the lung airways and interact with respiratory cells, where studies suggest they can induce inflammation, oxidative stress, and perturbations in lung function upon deposition in lower airways due to their small size and durability [70,71]. In contrast, inhaled house dust mite allergens, most of which are carried on fecal pellets and mite proteins, are recognized by the innate immune system as biologically active motifs that trigger airway epithelial signaling, recruit innate immune cells, and drive Th2-biased allergic inflammation characteristic of asthma and allergic rhinitis [72,73]. Human skin flakes and other occupant-derived particles contribute to indoor bioaerosols and can carry organic matter that influences indoor air chemistry and particulate composition, but they lack the defined allergenic proteins of house dust mite feces and the chemical persistence of synthetic microplastics [70,72]. These distinctions highlight that while all such particles share inhalation exposure routes, their biological interactions and immune consequences differ based on composition, immunogenic potential, and retention within the respiratory tract, underscoring the need for mechanistic research to distinguish microplastic effects from those of organism-derived aerosols.

Human exposure to plastic pollutants is commonly monitored through urine levels, but, due to the widespread endocrine-disrupting effects of these pollutants, human breast milk is an important medium to monitor for the presence of these chemicals. DEHP and its metabolites, primarily MEHP, have been detected globally in breast milk, with MEHP concentrations ranging from 8.3 nmol/L to 1.3 nmol/L. Some evidence shows that the concentration of specific phthalates like DINP may differ between geographic locations. This variation is due to a variety of factors, such as time of sampling and the local regulations surrounding phthalate use. Concentrations of phthalates in breast milk are not as significant nor do they correlate with urinary concentrations, so urine remains the more reliable biomonitoring medium [74]. Emerging research also shows that BPA presence is common in breast milk as well, with separate studies in the US, Panama, and Turkey reporting 2.1 ng/mL, 1.5 ng/mL and 0.63 ng/mL of BPA detected, respectively [75,76,77]. The presence of PFAS in breast milk was investigated and found that in 5 global regions, PFAS concentration ranged from 77 ng/L to 35 ng/L [78]. Microplastics also represent a global burden in breast milk, with studies reporting 38% of collected breast milk samples positive for MPs in Thailand and 26 out of 34 milk samples positive for MPs in Italy [79,80]. The size of the detected MPs also varies in breast milk from 2–50 μM [80]. As emerging reports expand the knowledge of the varied endocrine, immune, and developmental effects of these plastic-derived pollutants, it is imperative to continue to expand the base of knowledge about their presence in human breast milk and how their presence may affect viral infection in the mother and child.

The accumulation of these plastic-derived pollutants in the food chain is another important monitoring medium. DEHP and butyl benzyl phthalate (BBP) showed potential for trophic magnification in the mangrove ecosystem, while other phthalates did not [81]. In conjunction, multiple studies have shown that long-chain phthalate acid esters do not bioaccumulate, but their fat-soluble metabolites may be stored in the fat tissues of animals long term [82]. BPA and other Bisphenols (BPs) are represented in the food chain with a high bioaccumulation capacity in the aquatic food chain and pose risks to the public. Marine fish were found to make up 43.59% of total BPs exposure in a Chinese coastal city with a hazard quotient of 3.67 (risk threshold = 1) [83]. PFAS are detected globally in the terrestrial food chain, with multiple studies from China and Italy reporting levels in cow, pork, and eggs. Additionally, PFAS represent a large bioaccumulative risk in the food chain and humans due to their high polarity and thermal resistance, thus termed “forever chemicals” [84]. MPs have been detected widely in the food chain, with salt, seafood, and freshwater fish representing significant pathways into the human diet, but concentrations and risk of exposure differ between regions and are shaped by local pollution factors and food preparation techniques [85]. More research is needed to characterize the accumulation of these plastic-derived pollutants in the food chain, as current research implies that these chemicals are widely present in the global food web.

This review further suggests that phthalates such as DEHP and MEHP act through overlapping mechanisms, including redox imbalance, metabolic reprogramming, and microbiota-mediated modulation to influence immune health across the lifespan [5,24]. While the available evidence is compelling, direct human data linking specific body burdens to worse outcomes for defined viral infections remain limited. Future research should combine biomonitoring of phthalate metabolites with infection outcomes, alongside experimental validation of pathways such as IL-23 suppression and PPARγ-mediated dendritic cell changes, across multiple viral pathogens. In the interim, precautionary reduction in phthalate exposure, particularly in vulnerable groups such as pregnant women and infants, represents a pragmatic public health strategy.

Importantly, not all phthalates act uniformly as viral enhancers. For example, dibutyl phthalate (DBP) exacerbates HBV progression, while certain cyclic and polymeric phthalates such as cellulose acetate phthalate (CAP), hydroxypropyl methylcellulose phthalate (HPMCP), and polyvinyl acetate phthalate (PVAP) have demonstrated antiviral activity when used alone or in conjunction with existing therapeutics. Natural extracts containing phthalate-like compounds also show promise: the ethanolic extract of Holothuria parva (sea cucumber), which includes diisooctyl phthalate (DIOP), reduced HSV-1 infection, and increased host cell viability in vitro [86]. These divergent findings highlight a critical knowledge gap: while phthalate-based plasticizers in the environment can promote viral infections, structurally related phthalate derivatives may hold potential as therapeutic antivirals. This duality shows the need for nuanced research into phthalate chemistry, environmental exposure, and drug development potential.

## 12. Knowledge Gaps

Despite emerging evidence, several gaps remain. First, the majority of studies are experimental or ecological, and rigorous longitudinal human cohort studies linking pollutant exposure to biomarkers with viral outcomes are rare. Moreover, the interplay between pollutants and the microbiome, a known modulator of immunity and viral susceptibility, remains underexplored. In addition, there is limited understanding of how mixtures of pollutants interact in real-world settings, where humans are exposed simultaneously to phthalates, BPA, PFAS, and MPs. The effects of pollutants also vary by polymer chemistry, size, aging state, and virus type. Thus, standardized comparative studies are needed to establish the mechanism of each pollutant in the pathogenesis of infections.

## 13. Future Directions

Addressing these gaps requires a multidisciplinary approach that integrates environmental toxicology, virology, and public health. Omics-based platforms (transcriptomics, lipidomics, proteomics) will be critical for mapping pollutant-induced reprogramming of host–virus interactions. Advanced imaging and single-cell technologies can help track pollutant–virus interactions at cellular and subcellular levels. From a translational perspective, identifying pollutant-sensitive pathways such as lysosomal function, autophagic flux, or interferon signaling offers opportunities for host-directed antivirals. Finally, global policy efforts to reduce plastic production and human exposure carry not only ecological but also infectious disease benefits, underscoring the need to integrate pollution and pandemic preparedness agendas.

## Figures and Tables

**Table 1:** Plastic-derived pollutants and their effects during viral infection: summarizing known pathways and evidence levels.

Author (Year)	Plastic Pollutant	Source/Origin/Use	Viral Target	Epidemiological/Mechanistic Finding	Study Population	In Vitro/Animal Models	Evidence Level
Lin et al. (2021) [5]	DEHP/ MEHP	Widely used plasticizer in PVC; leaches from medical devices, food packaging, and indoor air	DENV	MEHP promotes DENV infection by suppressing IL-23-mediated macrophage antiviral responses	89 human patients	Cell lines (Human monocyte-derived macrophages)	Cross-sectional and experimental (Causation)
Kordbacheh et al. (2024) [24]			CVB	DEHP exposure exacerbates CVB infection severity	-	Cell lines (HeLa cells, iPSC/iBECs, Caco-2s, HL-1s)	Experimental (Causation)
Zhu et al. (2025) [25]	DBP	Leaches from Plastic Packaging and phthalate-containing plastics can be found in indoor and outdoor microenvironments	HBV	DBP promotes T cell dysfunction, increases HBV replication, and exposure is correlated with HBV positivity factors	9 liver cancer patients with HBV	C57BL/6 J mice (Male)	Cross-sectional and experimental (Causation)
Saito et al. (2022) [26]	Cyclic phthalates	Identified from the chemical library, not generally found in the environment	HCV	Attenuated HCV viral production	-	Cell lines (Huh-7 cells)	Experimental (Association)
			SARS-CoV-2	Reduced viral RNA of SARS-CoV-2 while significantly reducing lipid droplet formation	-	Cell lines (VeroE6/TMPRSS2 cells)	Experimental (Association)
Uddin et al. (2013) [27]	CPHP **	*Acrostichum aureum* L. (Pteridaceae), a Bangladeshi mangrove fern	DENV2	Reduced viral titres in Vero and LLC-MK2 cells in post-infection, suggesting inhibition of viral replication	-	Cell lines (Vero and LLC-MK2 cells)	Experimental (Association)
			CHIKV	Reduced virus titre in post-infection, suggesting inhibition of viral replication	-	Cell lines (Vero and LLC-MK2 cells)	Experimental (Association)
			hPiV3	Reduced viral titres in Vero and LLC-MK2 cells in post- infection, suggesting inhibition of viral replication	-	Cell lines (Vero and LLC-MK2 cells)	Experimental (Association)
Uhm et al. (2022) [28]	BPA	Polycarbonate plastics and epoxy resins	HBV	Significantly less urinary BPA concentration in HBV vaccinated individuals	NHANES data (6134 participants)	-	Cross-sectional (Association)
Roy et al. (2012) [29]			IAV	Developmental exposure to BPA in adult mice resulted in less severe IAV infection.	-	C57BL/6 mice (Female)	Proespective experimental (Causation)
Bruno et al. (2019) [30]			CVB3	BPA interferes with cardiac inflammation by dysregulation of ERs in viral myocarditis	-	BALB/c mice (Female)	Proespective experimental (Causation)
Bulka et al. (2021) [31]	PFAS	Water repellant surfaces: non-stick cookware, food packaging, firefighting foams, cosmetics	Herpesvirus, Hepatitis C, HIV	Higher pathogen burden results in higher concentration in serum PFAS	NHANES data (8778 participants)	-	Cross-sectional (Association)
Post et al. (2024) [32]			IAV	Mixtures of PFAS differentially affect the adaptive immunity during IAV infection.	-	C57Bl/6 mice (Female)	Proespective experimental (Causation)
Perez et al. (2023) [33]			TMEV	PFOA exposure during TMEV infection resulted in an imbalance of cytokines with no interaction between virus and PFOA.	-	C57BL/6J mice (Male and Female)	Experimental (Association)
Rhea et al. (2026) [34]			SARS-CoV-2	No consistent evidence linking higher serum PFAS to lower COVID-19 vaccine response	330 COVID-19-vaccinated adults	-	Cross-sectional (Association)
Wang et al. (2023) [21]	Microplastics	Cosmetic microbeads, industrial pellets or by fragmentation and abrasion of larger plastics	H1N1 influenza A virus (IAV)	MPs decreased antiviral defenses, including the interferon-inducible transmembrane protein IFITM3 and upstream RIG-I/IRF3/TBK1 signaling	-	Cell lines (A549 cells)	Experimental (Association)
Amato-Lourenco et al. (2021) [22]			SARS-CoV-2, Hepatitis B virus (HBV)	Acts as physical carriers for viruses, stabilizing viral genome, promoting viral attachment and host cell uptake	Pulmonary tissue from 20 nonsmoking adults	-	Simulations (Association)

Note: ** CPHP is not a plastic-derived pollutant, but its similarity in chemical structure to phthalates makes its effects on viral infections relevant. Additionally, CPHP is one of the few phthalic acid derivatives that display divergent negative effects on viral infection.

## Data Availability

No new data were created or analyzed in this study.

## Abbreviations

Di(2-ethylhexyl) phthalate (DEHP); mono(2-ethylhexyl) phthalate (MEHP); Dengue virus (DENV); Dibutyl phthalate (DBP); 2″-(methoxycarbonyl) 5″-methylpentyl 2′-methylhexyl phthalate (CPHP), Human Cytomegalovirus (HCMV), Epstein–Barr Virus (EBV), Herpes Simplex Virus type 1 (HSV-1).
